# Supplementary material for: Therapeutic Efficacy of Weissella cibaria CMU and CMS1 on Allergic Inflammation Exacerbated by Diesel Exhaust Particulate Matter in a Murine Asthma Model
Source: Medicina (Kaunas). 2022 Sep 19;58(9):1310. doi: 10.3390/medicina58091310 (PMC9500616; doi:10.3390/medicina58091310)
Supplement: Supplementary file 1 [file medicina-58-01310-s001.zip › medicina-1859135-supplementary.pdf]

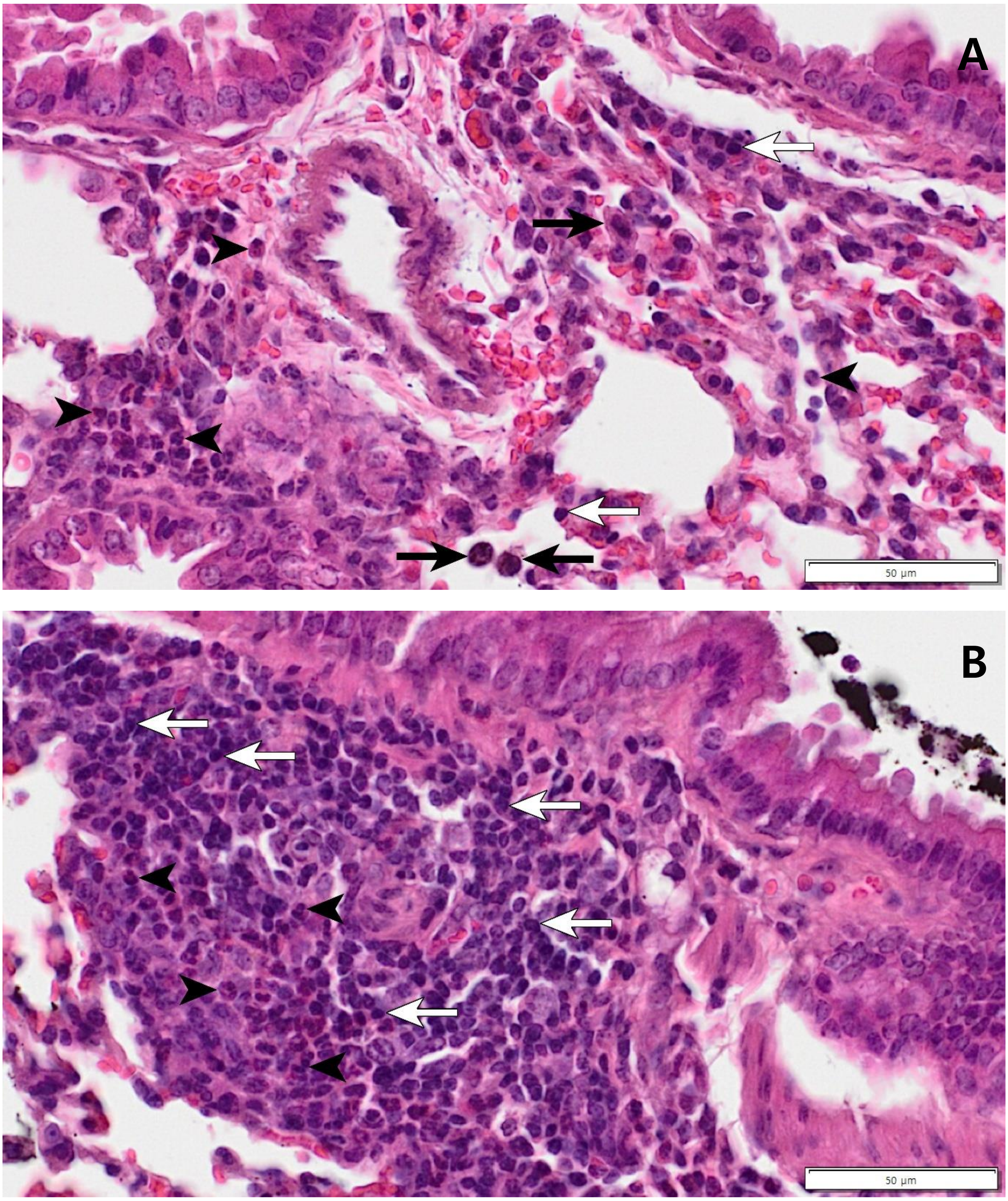

Supplementary Figure S1. Identification of inflammatory cells in higher magnification image of (**A**) CMS1-high and (**B**) PC group. Black arrows indicate macrophages, open arrows indicate lymphocytes, and arrow heads indicate eosinophilic infiltration. Scale bars = 50 μm.
